# The triglyceride glucose (TyG) index is associated with decreased myocardial mechano‐energetic efficiency in individuals with different glucose tolerance status

**DOI:** 10.1111/eci.70013

**Published:** 2025-02-25

**Authors:** Chiara M. A. Cefalo, Alessia Riccio, Teresa Vanessa Fiorentino, Elena Succurro, Maria Perticone, Velia Cassano, Angela Sciacqua, Francesco Andreozzi, Giorgio Sesti

**Affiliations:** ^1^ Department of Clinical and Molecular Medicine University of Rome‐Sapienza Rome Italy; ^2^ Department of Medical and Surgical Sciences University Magna Graecia of Catanzaro Catanzaro Italy

**Keywords:** cardiac energetics, cardiovascular disease, impaired glucose tolerance, insulin‐resistance, mechano‐energetic efficiency

## Abstract

**Background:**

This investigation had two main objectives: (1) to compare the triglyceride–glucose (TyG) index with the homeostasis model assessment of insulin resistance (HOMA‐IR) in relation to insulin‐stimulated myocardial glucose metabolic rate (MrGlu), measured by a dynamic positron emission tomography (PET) scan using 18F‐fluorodeoxyglucose (18F‐FDG) coupled with a euglycemic–hyperinsulinemic clamp; and (2) to assess whether the TyG index correlates with myocardial mechano‐energetic efficiency (MEE).

**Methods:**

We evaluated MrGlu in 46 participants who had no prior diagnosis of coronary heart disease. Myocardial MrGlu was quantified by 18F‐FDG PET during a euglycemic–hyperinsulinemic clamp. In a larger cohort of 1820 individuals, myocardial MEE per gram of left ventricular mass (MEEi) was measured echocardiographically. The TyG index was computed as the Ln [fasting triglycerides (mg/dL) × fasting glucose (mg/dL)/2].

**Results:**

When compared to HOMA‐IR, the TyG index exhibited a stronger correlation with myocardial MrGlu (Pearson's *r* = −.566 for TyG vs. –.471 for HOMA‐IR). Within the larger cohort, individuals in the highest TyG quartile showed significantly reduced MEEi compared to those in the lowest quartile (*p* < .001). Stepwise multivariate linear regression confirmed that the TyG index was the most significant determinant of MEEi, independent of traditional cardio‐metabolic risk factors.

**Conclusions:**

Our findings suggest that the TyG index is superior to HOMA‐IR as an indicator of cardiac insulin resistance and that it independently correlates with MEEi. Thus, the TyG index may serve as a valuable, readily available tool to identify subjects at elevated cardiovascular risk.

## Graphical abstract

A higher TyG index can identify individuals at increased cardiovascular risk.

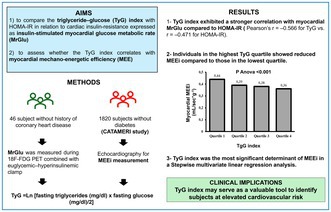

## 1 INTRODUCTION

Disturbances in myocardial energetics, reflected by diminished left ventricular (LV) mechanical efficiency and increased oxygen requirements, have been implicated in the pathogenesis of cardiovascular (CV) disorders.^1^, ^2^, ^3^, ^4^, ^5^, ^6^, ^7^ LV function depends principally on aerobic metabolic pathways, making the balance between LV performance and myocardial oxygen consumption (MVO_2_) crucial for cardiac efficiency.^8^ Mechano‐energetic efficiency (MEE) expresses the proportion of oxygen used to generate external mechanical work (i.e., stroke work) normalized to the oxygen consumed per contraction.^1^, ^2^, ^8^

Although direct quantification of MVO_2_ using approaches like coronary sinus catheterization^9^ or noninvasive PET scans^10^ can be accurate, these methods are cumbersome, expensive, and generally impractical for large‐scale clinical or epidemiological utilization. Hence, a more accessible, ultrasound‐based method has been introduced to estimate myocardial MEE, computed as the ratio of LV stroke work (stroke volume ×\times systolic blood pressure) to MVO_2_, the latter approximated by the product of systolic blood pressure and heart rate (often called the ‘double product’).^1^, ^2^, ^11^, ^12^ Clinical evidence links reduced MEE to conditions commonly characterized by insulin resistance, such as obesity, metabolic syndrome, impaired glucose regulation and type 2 diabetes.^13^, ^14^, ^15^, ^16^, ^17^ One plausible mechanistic contributor to changes in myocardial MEE is cardiac insulin resistance. Previous data indicate that depressed myocardial insulin sensitivity, measured by dynamic 18F‐FDG PET with euglycemic–hyperinsulinemic clamp, is accompanied by a lower echocardiographic estimate of MEE in individuals with varying degrees of glucose tolerance.^18^

While 18F‐FDG PET combined with a euglycemic–hyperinsulinemic clamp offers a robust measurement of myocardial insulin sensitivity, its complexity and expense render it ill‐suited to routine clinical practice or widespread research screenings. To address this, simpler surrogate markers have been developed. Among these, the triglyceride–glucose (TyG) index, which uses fasting glucose and triglycerides—two readily available clinical measures—has emerged as a plausible indicator of insulin resistance.^19^, ^20^ This index demonstrates a close relationship with insulin‐stimulated whole‐body glucose disposal, as measured by the clamp technique.^19^, ^20^, ^21^

Despite this evidence, whether the TyG index specifically tracks cardiac insulin sensitivity remains uncertain. Furthermore, its relationship with the myocardial MEE remains uncharted. Therefore, our study aimed to^1^ compare the TyG index and the well‐known HOMA‐IR index in predicting myocardial insulin resistance, determined by insulin‐stimulated myocardial MrGlu via 18F‐FDG PET and a euglycemic–hyperinsulinemic clamp, and^2^ examine the association of TyG with myocardial MEE in adults with varying levels of glucose tolerance participating in the CATAnzaro MEtabolic RIsk factors (CATAMERI) study.^13^, ^14^, ^17^

## 2 PATIENTS AND METHODS

The study was approved by the local institutional review board (Comitato Etico Azienda Ospedaliera ‘Mater Domini’), and all participants provided written informed consent in accordance with the Declaration of Helsinki.

### 2.1 Study population

#### 2.1.1 Sample 1

This group consisted of 46 adults with varying glycemic statuses enrolled at the ‘Magna Graecia’ University of Catanzaro.^22^ Among those with type 2 diabetes, only individuals on a stable metformin dose (≥1500 mg/day) were included. All participants underwent anthropometric examinations (height, weight, body mass index [BMI] and waist circumference) and blood pressure measurements. A 75‐g oral glucose tolerance test (OGTT) was performed in individuals with fasting plasma glucose <126 mg/dL (7.0 mmol/L), HbA1c <6.5% (48 mmol/mol), and no prior history of diabetes. On a separate day, participants underwent^1^ echocardiography to measure MEEi and^2^ 18F‐FDG PET scans under euglycemic–hyperinsulinemic clamp conditions to determine myocardial insulin sensitivity.^18^, ^22^, ^23^

#### 2.1.2 Sample 2

The second cohort included 1820 white adults from the CATAMERI observational study. Subjects were eligible if they possessed at least one of the following risk factors: overweight/obesity, hyperglycemia, hypertension or dyslipidemia.^13^, ^14^, ^17^, ^24^ During the initial visit (following an overnight fast), anthropometric data—BMI, waist circumference and blood pressure—were collected, and venous blood samples were taken for laboratory tests. On a separate day, a 75‐g OGTT was carried out in individuals without a prior diagnosis of diabetes.

### 2.2 Echocardiographic measurements

All echocardiograms were performed by the same trained sonographer using a VIVID‐7 Pro ultrasound machine (GE Technologies, Milwaukee, WI) equipped with a 2.5‐MHz transducer. LV end‐diastolic volume (LVEDV), LV end‐systolic volume (LVESV) and LV ejection fraction (LVEF) were measured following the Simpson biplane rule.^25^ Stroke volume was computed as LVEDV—LVESV and then normalized by height for a stroke index. Myocardial MEE was calculated based on DeSimone and colleagues' ultrasound‐based formula.^1^, ^2^, ^12^, ^15^, ^16^ This approach estimates external myocardial work via stroke work (systolic blood pressure × times stroke volume) and approximates myocardial oxygen consumption using the product of systolic blood pressure and heart rate. Finally, MEE was standardized to LV mass (indexed MEE, or MEEi) to account for LV mass variability.^2^, ^12^, ^15^

### 2.3 Laboratory measurements

Glucose, triglycerides, total and high‐density lipoprotein (HDL) cholesterol were measured by enzymatic assays (Roche, Basel, Switzerland). Insulin levels were quantified using a chemiluminescence immunoassay (Immulite®, Siemens Healthcare GmbH, Erlangen, Germany). High‐sensitivity C‐reactive protein (hs‐CRP) was measured on a BN™II System (Siemens Healthcare, Marburg, Germany). Serum uric acid was determined using the URICASE/POD method (Boehringer Mannheim, Mannheim, Germany).

### 2.4 Calculations

Glucose tolerance status was characterized by the American Diabetes Association criteria using fasting and 2‐h post‐OGTT plasma glucose values.^26^ Individuals were categorized into normal glucose tolerance (NGT), isolated impaired fasting glucose (IFG), impaired glucose tolerance (IGT) or type 2 diabetes.

The homeostasis model assessment index of insulin resistance (HOMA‐IR) was defined as fasting insulin (microU/L) × fasting glucose (mg/dL)/22.5.^27^ The TyG index was calculated as the Ln [fasting triglycerides (mg/dL) × fasting glucose (mg/dL)/2].^19^

### 2.5 Statistical analysis

Variables with skewed distributions (e.g., triglycerides, hs‐CRP, and insulin) were log‐transformed. Continuous variables are presented as mean ± standard deviation (SD). Categorical variables were compared using the chi‐square test. A general linear model with Fisher's least significant difference post hoc adjustments was applied to evaluate differences across groups, controlling for potential confounders. Correlations involving MEEi and clinical traits were assessed by Pearson's or Spearman's coefficients, depending on data distribution. A stepwise multivariate linear regression was then conducted to identify independent predictors of MEEi.To gauge how effectively the TyG index distinguishes individuals with high cardiac insulin resistance from those without, we employed a receiver operating characteristic (ROC) curve analysis. Cardiac insulin resistance was defined as being in the lowest tertile of insulin‐stimulated myocardial MrGlu. Previous studies have shown a 28%–50% decrease in insulin‐stimulated myocardial glucose uptake in subjects with varying degrees of insulin resistance, such as prediabetes and type 2 diabetes.^28^ In light of this, using an online power calculator (https://clincalc.com/Stats/SampleSize.aspx), we calculated that 15 individuals in each group would have 90% power to detect a 28% difference in myocardial glucose uptake, with a significance level of 5%. Significance was set at *p* ≤ .05. Statistical calculations were carried out using SPSS version 27 for Windows (IBM Corp., Armonk, NY, USA).

## 3 RESULTS

### 3.1 Cross‐sectional analysis in Sample 1

**FIGURE 1:**
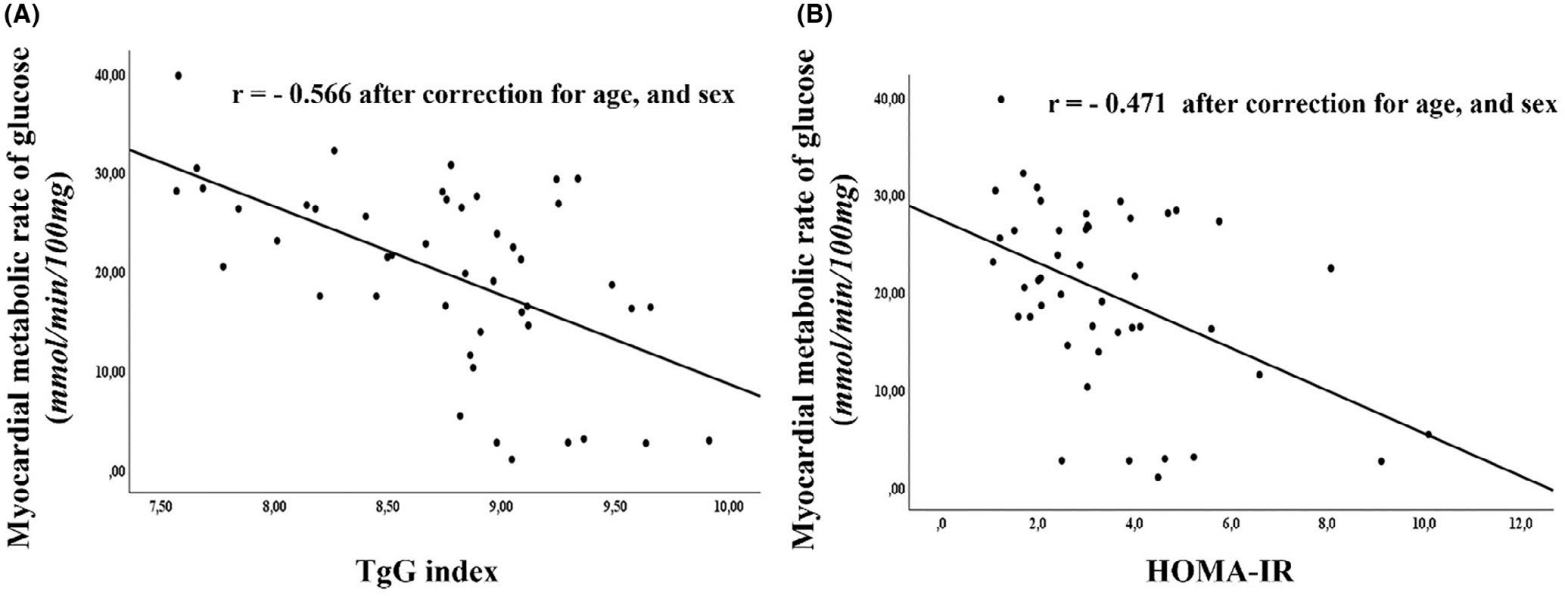
Association between insulin‐stimulated myocardial glucose metabolic rate (MrGlu) and TyG index (panel A) or HOMA‐IR index (panel B). MrGlu has been assessed by a 18F‐FDG PET scan combined with euglycemic hyperinsulinemic clamp in 46 subjects with different conditions of glucose intolerance. The triglyceride glucose (TyG) index was calculated as the Ln [fasting triglycerides (mg/dL) × fasting glucose (mg/dL)/2]. The homeostasis model assessment index of insulin resistance (HOMA‐IR) was defined as fasting insulin (microU/L) × fasting glucose (mg/dL)/22.5. *r* = Pearson correlation coefficient.

**FIGURE 2:**
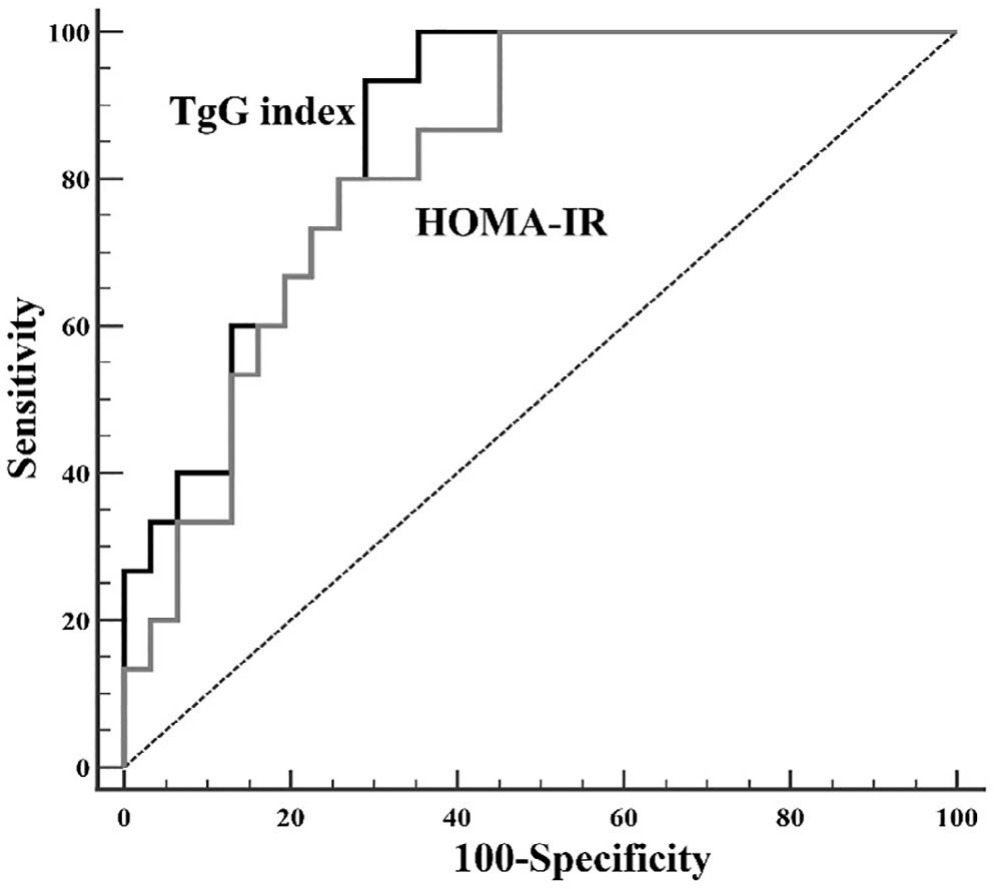
ROC curves comparing the accuracy of the TyG index and the HOMA‐IR index in identifying cardiac insulin resistance. The accuracy of each index is assessed using the area under the curve (AUC), which quantifies the overall diagnostic performance. A higher AUC value indicates a better ability to discriminate between individuals with and without cardiac insulin resistance in sample 1. HOMA‐IR, homeostasis model of insulin resistance; ROC, receiver operating characteristic; TyG, triglyceride‐glucose.

Table 1 summarizes the baseline anthropometric and metabolic attributes of the 46 participants, of whom 16 (34.8%) had NGT, 10 (21.7%) had prediabetes and 20 (43.5%) had type 2 diabetes. The TyG index significantly correlates with HOMA‐IR (*r* = .391, Figure S1). Pearson's correlation revealed that insulin‐stimulated myocardial MrGlu had a stronger negative association with the TyG index (*r* = −.566, Figure 1A) than with HOMA‐IR (*r* = −.471, Figure 1B). Using ROC analysis to assess the diagnostic accuracy of TyG for cardiac insulin resistance (defined as the lowest tertile of myocardial MrGlu), we found an AUC of .860 (95% CI: .757–.963), demonstrating that TyG accurately discerns individuals with elevated cardiac insulin resistance (Figure 2).

**TABLE 1:** Anthropometric and metabolic characteristics of sample 1 participants (*n* = 46).

Variables	Parameters
Sex (men/women)	23/23
Age (yrs)	51 ± 9
BMI (kg/m^2^)	29.8 ± 4.9
Waist circumference (cm)	102 ± 12
Systolic blood pressure (mmHg)	122 ± 15
Diastolic blood pressure (mmHg)	76 ± 10
Fasting glucose (mg/dL)	110 ± 29
2‐h glucose (mg/dL)	127 ± 22
Fasting plasma insulin (μUI/mL)	13 ± 8
Glucose tolerance status (NGT/Prediabetes/ T2DM) (number)	16/10/20
Total cholesterol (mg/dL)	191 ± 40
HDL (mg/dL)	47 ± 11
Triglycerides (mg/dL)	131 ± 62
HOMA‐IR index	3.4 ± 2.0
TyG index	8.7 ± .5
Myocardial MRGlu (μmol/min/100 g)	19.8 ± 9.2

*Note*: Data are means ± SD.

Abbreviations: BMI, body mass index; HOMA‐IR, homeostasis model assessment for insulin resistance; NGT, normal glucose tolerance; Prediabetes, impaired fasting glucose and/or impaired glucose tolerance; T2DM, type 2 diabetes.

### 3.2 Cross‐sectional analysis in Sample 2

In the larger cohort (*N* = 1820; mean age 49 ± 14 years, 43% female), participants were categorized by quartiles of the TyG index (Table 2). Higher TyG quartiles tended to include older individuals, a more significant proportion of males, more current smokers, and higher BMI and waist circumference values than lower quartiles. Additionally, fasting and 2‐h post‐load glucose, insulin levels, total cholesterol, triglycerides, uric acid and hs‐CRP were elevated, while HDL cholesterol was lower in the top quartiles of TyG. These participants also more frequently exhibited impaired glucose homeostasis.

**TABLE 2:** Anthropometric and metabolic characteristics of sample 2 participants stratified according to TgG index quartiles.

Variables	Quartile 1	Quartile 2	Quartile 3	Quartile 4	*p*
	*n* = 456	*n* = 454	*n* = 455	*n* = 455	
Sex (male/female)	142/314	197/257^a^	247/208^a^, ^d^	279/176^a^, ^c^, ^f^	<.001
Age (years)	42 ± 13	50 ± 12^a^	51 ± 12^a^	52 ± 11^a^	<.001^§^
BMI (kg/m^2^)	28.3 ± 6.3	30.7 ± 6.1^a^	30.8 ± 5.7^a^	31.6 ± 6.1^a^, ^d^, ^f^	<.001
Waist circumference (cm)	96 ± 14	103 ± 13^a^	103 ± 13^a^	106 ± 13^a^, ^d^, ^f^	<.001
Smoking status (never smokers/current smokers/ex‐smokers) (number)	292/101/63	255/84/115^a^	251/92/112^a^	210/116/129^a^, ^d^, ^f^	<.001
Systolic blood pressure (mmHg)	122 ± 16	132 ± 17^a^	132 ± 16^a^	134 ± 15^a^	<.001
Diastolic blood pressure (mmHg)	76 ± 11	81 ± 10^a^	81 ± 10^a^	83 ± 10^a^	<.001
Total cholesterol (mg/dL)	182 ± 33	195 ± 37^a^	206 ± 36^a^, ^c^	216 ± 40^a^, ^c^, ^e^	<.001
HDL (mg/dL)	59 ± 15	51 ± 11^a^	48 ± 11^a^, ^c^	43 ± 12^a^, ^c^, ^e^	<.001
Triglycerides (mg/dL)	64 ± 13	97 ± 14a	132 ± 20^a^, ^c^	220 ± 87^a^, ^c^, ^e^	<.001
Fasting glucose (mg/dL)	86 ± 9	93 ± 9^a^	96 ± 11^a^, ^c^	102 ± 15^a^, ^c^, ^e^	<.001
2 h post load glucose (mg/dL)	111 ± 32	126 ± 38^a^	133 ± 43^a^, ^c^	150 ± 48^a^, ^c^, ^e^	<.001
Fasting insulin (μU/mL)	10 ± 7	13 ± 8^a^	15 ± 9^a^, ^d^	16 ± 10^a^, ^c^, ^e^	<.001
Uric acid (mg/dL)	4.3 ± 1.2	5.1 ± 1.2^a^	5.3 ± 1.3^a^	5.7 ± 1.3^a^, ^c^, ^e^	<.001
hs‐CRP (mg/L)	3.2 ± 3.2	4.0 ± 3.9^a^	4.2 ± 4.1^b^	4.4 ± 4.2^a^	.006
TgG index (range)	7.9 ± .23 (6.66–8.22)	8.4 ± .10^a^ (8.23–8.59)	8.7 ± .09^a^, ^c^ (8.60–8.93)	9.2 ± .30^a^, ^c^, ^f^ (8.93–10.87	<.001
Glucose tolerance (NGT/IFG/IGT/IFG‐IGT/new T2DM)	359/22/55/11/9	272/47/69/41/25^a^	214/79/62/59/41^a^, ^c^	138/88/61/87/81^a^, ^c^, ^e^	<.001

*Note*: Data are expressed as mean ± SD. Triglycerides, hs‐CRP and fasting insulin levels were log transformed for statistical analysis, but values in the table represent back transformation to the original scale. Comparisons between the four groups were performed using a general linear model with post hoc Fisher's least significant difference correction for pairwise comparisons. ^§^ *p* values refer to results after analyses with adjustment for sex and age.

Abbreviations: BMI, body mass index; HDL, high density lipoprotein; hs‐CRP, high sensitive C reactive protein; IFG, impaired fasting glucose; IGT, impaired glucose tolerance; new T2DM, newly diagnosed type 2 diabetes mellitus; NGT, normal glucose tolerance; TyG index, triglyceride glucose index.

^a^ *p* < .05 versus Quartile 1.

^b^ *p* < .001 versus Quartile 2.

^c^ *p* < .05 versus Quartile 2.

^d^ *p* < .001 versus Quartile 3.

^e^ *p* < .05 versus Quartile 3.

^f^ *p* <.001 versus Quartile 1.

^g^ *p* values refer to results after analyses with adjustment for sex.

**FIGURE 3:**
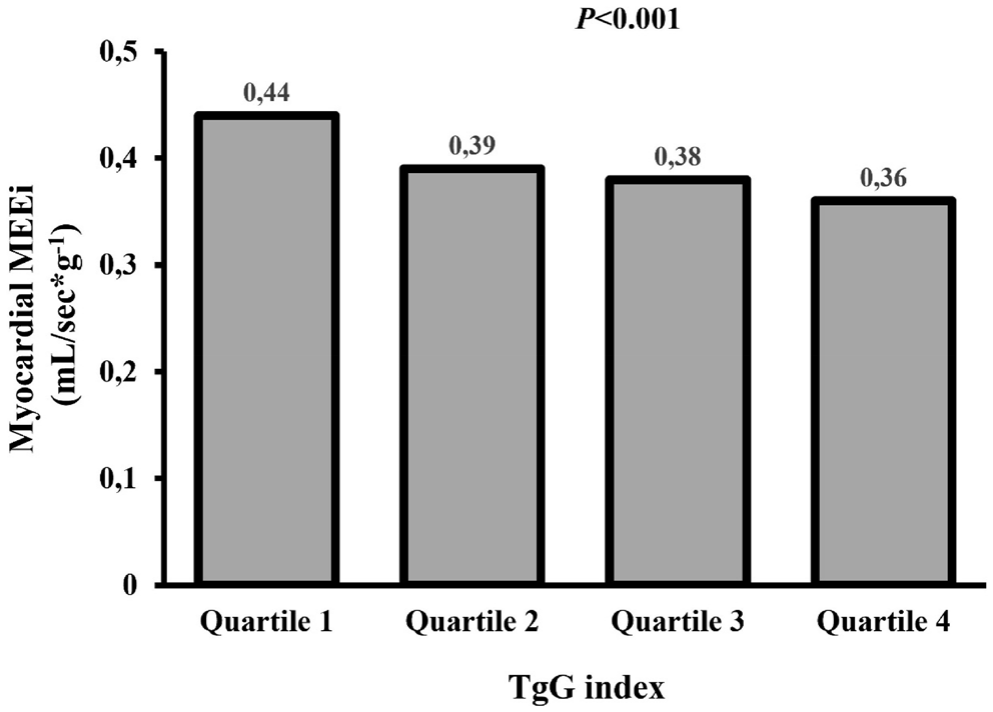
Myocardial MEEi values of the study population stratified according to TyG index quartiles. Myocardial MEEi has been calculated as the ratio between stroke work and myocardial oxygen consumption, while the TyG index was calculated as the Ln [fasting triglycerides (mg/dL) × fasting glucose (mg/dL)/2] in 1280 subjects included in Sample 2. Quartile 1: TyG values range from 6.66 to 8.22, quartile 2: TyG values range from 8.23 to 8.59, quartile 3: TyG values range from 8.60 to 8.93, quartile 4: TyG values range from 8.93 to 10.87. MEEi, mechano‐energetic efficiency per gram of left ventricular mass; TyG, triglyceride‐glucose. *p*‐value, comparison among groups.

Echocardiographic evaluations (Table 3) revealed that elevated TyG was accompanied by a higher LV mass index (after adjusting for age and sex), heart rate, stroke work and myocardial oxygen consumption, as well as noticeably reduced MEEi compared to the lowest quartile (Figure 3, Table 3).

**TABLE 3:** Echocardiographic parameters of the study cohort stratified according to TgG index quartiles.

Variables	Quartile 1	Quartile 2	Quartile 3	Quartile 4	*p*
	*n* = 456	*n* = 454	*n* = 455	*n* = 455	
LV end‐systolic volume (mL)	23.8 ± 15.4	35.17 ± 17.0	37.4 ± 22.9	37.7 ± 22.1	.81
LV end‐diastolic volume (mL)	117 ± 37	122 ± 38	126 ± 41	126 ± 40	.89
LVM (g)	174 ± 59	202 ± 61^d^	210 ± 63^d^	218 ± 61^d^	<.001
LVM index (g/m^2.7^)	94 ± 24	105 ± 28	109 ± 30^a^	112 ± 29^a^	.04
Stroke volume (mL)	84 ± 26	87 ± 25	89 ± 25	89 ± 25	.68
Stroke work (mmHg*mL)	10,441 ± 3780	11,631 ± 3790^a^	11,778 ± 3794^a^	12,010 ± 3805^a^	.04
Heart rate (bpm)	69 ± 10	71 ± 10^a^	71 ± 10^a^	71 ± 10^a^	.009
Myocardial oxygen consumption (mmHg × bpm)	8661 ± 1803	9388 ± 1910^d^	9371 ± 1913^d^	9571 ± 1817^d^	<.001
Myocardial MEEi (mL/sec*g^−1^)	.44 ± .12	.39 ± .11^d^	.38 ± .11^d^	.36 ± .09^a^, ^b^, ^c^	<.001

*Note*: Data are means ± SD. Comparisons between the three groups were performed using a general linear model with post hoc Fisher's least significant difference correction for pairwise comparisons. *p* values refer to results after analyses with adjustment for sex and age.

Abbreviations: LV, left ventricular; LVM, left ventricular mass; LVMI, left ventricular mass index; MEEi, LVM‐normalized mechano‐energetic efficiency.

^a^ *p* < .05 versus Quartile 1.

^b^ *p* < .001 versus Quartile 2.

^**c**^ *p* < .05 versus Quartile 3.

^d^ *p* <.001 versus Quartile 1.

To identify clinical correlates of MEEi, we performed a univariate analysis (Table 4). MEEi was inversely related to age, male sex, BMI, waist circumference, smoking, total cholesterol, triglycerides, uric acid, hs‐CRP, fasting insulin, HOMA‐IR, TyG and abnormal glucose tolerance; conversely, it was positively related to HDL levels. Subsequently, these variables were entered into a stepwise multivariate regression (Table 5). The TyG index emerged as the most potent contributor to MEEi, explaining 7.9% of MEEi variability (*p* < .001). Age and fasting insulin levels accounted for 2.4% (*p* < .001), while sex, glucose tolerance category and hs‐CRP contributed 1.1%, .5% and .3%, respectively. Notably, adding HOMA‐IR to the model did not modify the significance of TyG, underscoring TyG's stronger link to MEEi.

**TABLE 4:** Univariate analysis between myocardial MEEi and cardio‐metabolic parameters.

Variables	Myocardial MEEi	*p*
	*R*	
Sex^a^	−.16	<.001
Age (years)	−.20	<.001
BMI (kg/m^2^)	−.15	<.001
Waist circumference (cm)	−.20	<.001
Smoking status (never smokers/current smokers/ex‐smokers)^a^	−.05	.03
Total cholesterol (mg/dL)	−.10	<.001
HDL‐C (mg/dL)	.14	<.001
Triglycerides (mg/dL)	−.22	<.001
Glucose tolerance status^a^	−.25	<.001
Fasting insulin (μU/mL)	−.24	<.001
Uric acid (mg/dL)	−.19	<.001
hs‐CRP (mg/L)	−.10	<.001
HOMA‐IR index	−.23	<.001
TgG index	−.26	<.001

Abbreviations: BMI, body mass index; HDL, high density lipoprotein, hs‐CRP, high sensitive C reactive protein; MEEi, LVM‐normalized mechano‐energetic efficiency; TyG index, triglyceride glucose index.

^a^ Spearman coefficient.

**TABLE 5:** Stepwise multiple regression analysis with MEEi as the dependent variable.

	Myocardial MEEi		
Variables	Total *R* ^2^ (%)	Partial *R* ^2^ (%)	*p*
TgG index	7.9	7.9	<.001
Fasting insulin (μU/mL)	10.9	3.0	<.001
Age (years)	13.2	2.3	<.001
Sex	14.3	1.1	<.001
Glucose tolerance status	14.8	.5	.005
Total cholesterol (mg/L)	15.1	.3	.04

*Note*: Independent variables associated to MEEi after stepwise regression analysis in a model including sex, age, BMI, smoking status, total cholesterol, HDL‐C, glucose tolerance status, fasting insulin, uric acid and hs‐CRP.

Abbreviations: BMI, body mass index; HDL, high density lipoprotein; hs‐CRP, high sensitive‐C reactive protein; MEEi, LVM‐normalized mechano‐energetic efficiency; TyG index, triglyceride glucose index.

## 4 DISCUSSION

A key finding of this investigation is that the TyG index correlates more strongly with myocardial insulin resistance, measured by 18F‐FDG PET under euglycemic–hyperinsulinemic conditions, than the commonly used HOMA‐IR. The ROC analysis supported the utility of TyG in detecting high cardiac insulin resistance, showing an AUC of .860 for identifying individuals in the bottom tertile of myocardial MrGlu. Additionally, higher TyG levels were independently linked with lower myocardial MEEi, an emergent cardiovascular risk marker.^1^, ^2^, ^6^, ^29^ Even after controlling for numerous traditional cardio‐metabolic risk factors (age, sex, BMI, smoking, total cholesterol, HDL cholesterol, fasting insulin, glucose tolerance status, hs‐CRP, uric acid and HOMA‐IR), the TyG index remained the principal determinant of MEEi, suggesting a direct effect of cardiac insulin resistance in promoting myocardial impairment. It is important to note that while the myocardium primarily relies on free fatty acids (FFA) as the main energy substrate under normal, resting conditions, glucose plays a crucial role in maintaining metabolic flexibility, enabling the heart to adapt to increased energy demands.^30^ In fact, during stress conditions such as ischemia, fatty acid oxidation decreases while glucose utilization increases. In states of insulin resistance, the ischemic heart is unable to increase glucose utilization, and energy production remains dependent on fatty acid oxidation. These changes result in increased myocardial oxygen consumption for a given workload, ultimately reducing cardiac efficiency and increasing oxidative stress. This, in turn, may lead to alterations in mechano‐energetic performance, left ventricular maladaptive changes, cardiac dysfunction, and ultimately contribute to the development of heart failure and coronary artery disease. Impaired myocardial energetics, as indicated by a reduced MEEi, has been associated with adverse cardiovascular events such as heart failure, myocardial infarction and other cardiovascular complications, all of which are known to impact mortality.^1^, ^2^, ^6^ However, future studies exploring the relationship between MEEi and long‐term mortality are required to determine the impact of depressed MEEi on both cardiovascular and all‐cause mortality.

These results support emerging evidence that the TyG index is a credible predictor of CV risk and an effective tool in preventive cardiology. Indeed, a recent meta‐analysis pooling eight cohort studies (5,731,294 individuals) reported that participants with the highest TyG index had a 61% greater hazard of atherosclerotic cardiovascular disease events compared to those with the lowest TyG values.^31^ Our data extend these findings specifically to myocardial insulin resistance, showing that TyG tracks well with gold‐standard measures and correlates with an important echocardiographic index of myocardial efficiency. The question of whether the relationship between MEEi and insulin resistance is a primary or secondary phenomenon remains unresolved. MEEi is defined as the ratio between the produced external work (measurable as stroke work, i.e., systolic blood pressure × stroke volume) and the amount of oxygen consumed during contraction, which is estimated as the product of heart rate and systolic blood pressure. It is well established that insulin resistance plays a pathogenic role in increasing both systolic blood pressure and heart rate through dysregulation of renal sodium metabolism, the renin‐angiotensin‐aldosterone system and the sympathetic nervous system. Therefore, it seems plausible that insulin resistance may precede and contribute to depressed MEEi. However, further longitudinal studies are needed to firmly establish the primary causal role of insulin resistance in myocardial energetics. HOMA‐IR has traditionally been the favoured surrogate for insulin resistance and is associated with reduced myocardial MEEi^13^, ^16^; however, its reliability diminishes when insulin secretory capacity is low or fasting glucose levels are high.^32^ In addition, fasting insulin measurements are not universally performed in clinical workflows, which limits HOMA‐IR's broader application. The TyG index, by contrast, is derived solely from fasting glucose and triglyceride values, which are routinely assessed in primary care settings and thus offers a more practical metric for screening and epidemiological projects. Thus, the TyG index can be a suitable tool for cardiovascular risk stratification in individuals who may benefit from early pharmacological and nonpharmacological interventions.

The present study has some strengths deserving consideration. One strength of the study is using the euglycemic–hyperinsulinemic clamp combined with 18F‐FDG PET for direct measurement of myocardial glucose uptake.^33^ This setup, while highly accurate, is rarely applied in large cohorts due to cost and complexity. Additionally, the association of the TyG index with MEE was investigated in a relatively large sample of adult individuals, including both men and women without a prior diagnosis of diabetes or cardiovascular disease, whose glucose tolerance was accurately assessed using fasting and 2‐h post‐load glucose levels during an OGTT. Moreover, in the larger sample, echocardiographic measurements were conducted by a single‐blinded operator, minimizing interobserver bias. Fresh blood samples were used for metabolic assays, further enhancing data quality.

Several limitations should be noted. First, myocardial MEEi was indirectly estimated rather than invasively measured via coronary sinus sampling. However, invasive techniques are generally not viable for population‐scale research. Second, the cross‐sectional design limits the ability to draw conclusions about causality between myocardial insulin resistance and compromised MEEi. Future studies using a longitudinal design are needed to evaluate the temporal relationship and causal links between these variables, as well as to further investigate the impact of insulin resistance and impaired cardiac energetics on clinical outcomes. Third, OGTTs were administered only once, which may have introduced some misclassification due to normal day‐to‐day variation in glucose metrics. Finally, our cohort, predominantly white outpatients from a specialised university hospital, may not be broadly representative of other populations or ethnicities.

## 5 CONCLUSION

The present study suggests that the TyG index is a better surrogate index of cardiac insulin resistance than the HOMA‐IR index, representing a valuable tool to identify individuals at higher cardiovascular risk. Future studies in more diverse populations and with longitudinal follow‐up could help clarify whether interventions to improve the TyG index also translate to better myocardial efficiency and CV outcomes.

## AUTHOR CONTRIBUTIONS

C.M.A.C. and A.R. contributed to conceive the study, critically reviewed and edited the manuscript; T.V.F., E.S., M.P., V.C., A.S. and F.A. collected data and reviewed the manuscript. G.S. designed the study, analysed the data and wrote the manuscript.

## FUNDING INFORMATION

This study was supported in part by grants from the Sapienza University of Rome n. RM1201728887461F and Italian Ministry of University n. 2020N5WK98_005 to G.S. T.V.F. was supported in part by the Mario Condorelli Award from the Italian Internal Medicine Society and the EFSD/Lilly Young Investigator Research Award from the European Foundation for the Study of Diabetes.

## CONFLICT OF INTEREST STATEMENT

The authors declare that they have no competing interests.

## Supporting information


Figure S1.

## Data Availability

The datasets used and analysed during the current study are available from the corresponding author on reasonable request.
