# Supplementary material for: The triglyceride glucose (TyG) index is associated with decreased myocardial mechano‐energetic efficiency in individuals with different glucose tolerance status
Source: Eur J Clin Invest. 2025 Feb 25;55(6):e70013. doi: 10.1111/eci.70013 (PMC12066897; doi:10.1111/eci.70013)
Supplement: Supplementary file 1 — Figure S1. [file ECI-55-e70013-s001.docx]

**Supplementary data**

**Figure 1. Association between TyG index and HOMA-IR index in sample 1.** The triglyceride glucose (TyG) index was calculated as the Ln [fasting triglycerides (mg/dl) x fasting glucose (mg/dl)/2]. The homeostasis model assessment index of insulin resistance (HOMA-IR) was defined as fasting insulin (microU/L) x fasting glucose (mg/dl)/22.5. Sample 1 comprises 46 subjects without cardiovascular diseases and different conditions of glucose intolerance. r= Pearson correlation coefficient.

**
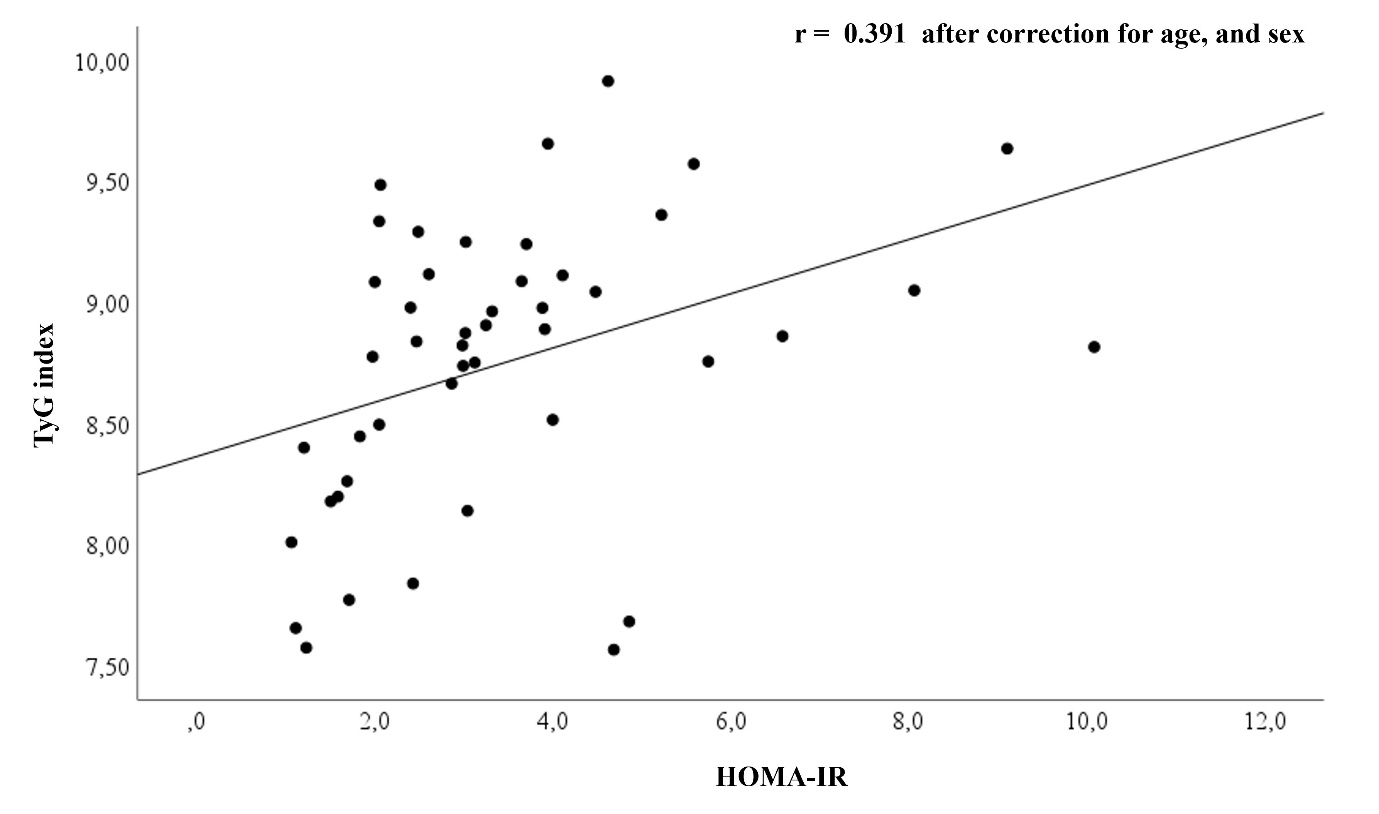
**
